# Effect of 5-weeks participation in The Daily Mile on cognitive function, physical fitness, and body composition in children

**DOI:** 10.1038/s41598-022-18371-w

**Published:** 2022-08-22

**Authors:** Karah J. Dring, Lorna M. Hatch, Ryan A. Williams, John G. Morris, Caroline Sunderland, Mary E. Nevill, Simon B. Cooper

**Affiliations:** Sport Health and Performance Enhancement (SHAPE) Research Group, Department of Sport Science, School of Science and Technology, Nottingham Trent University, Clifton Campus, Nottingham, NG11 8NS UK

**Keywords:** Risk factors, Paediatrics, Public health, Weight management

## Abstract

The aim of the present study was to determine the effect of 5-weeks participation in The Daily Mile on cognitive function, physical fitness, and adiposity in primary school children. In a quasi-experimental study, one class from each school completed The Daily Mile (n = 44) or acted as a control group (n = 35). Baseline measures included cognitive function tests (Stroop test, Sternberg paradigm, Flanker task), physical fitness (multi-stage fitness test) and body composition (BMI percentile, waist:hip circumference, sum of skinfolds). The intervention group completed 5-weeks of The Daily Mile. Follow-up measurements were completed within 48-h of the last training session. Data were analysed via ANCOVA, examining between group differences at follow-up, controlling for baseline values. Response times on the complex Stroop test were faster at follow-up in the intervention group (Intervention: 1357 ms [1280–1400 ms]; Control: 1463 ms, [1410–1523 ms], *d* = 0.31*, p* = 0.048). There was no effect of The Daily Mile on the Sternberg paradigm or Flanker test. Physical fitness was greater at follow-up in the intervention group (Intervention: 880 m, [820–940 m]; Control: 740 m, [680–800 m], *d* = 0.39, *p* = 0.002). There was no effect of the intervention on adiposity. In conclusion, five-weeks of The Daily Mile enhanced inhibitory control and physical fitness in children, but did not affect working memory, attention, or adiposity.

## Introduction

Children and adolescents in the United Kingdom (UK) are recommended to participate in an average of 60-min moderate-to-vigorous physical activity each day^1^; however, 77% of boys and 80% of girls aged 5–15 years fail to meet these recommendations^2^. Such low physical activity levels are associated with reduced physical fitness and increased adiposity^3^. This is of concern given that low physical fitness and increased adiposity are associated with increased risk factors for cardiometabolic diseases^4^ and impairments in cognitive function^5^, with detriments in cognition associated with lower academic achievement in children and adolescents^6,7^.

As children and adolescents spend a significant proportion of awake time at school, UK public health guidance states that 30-min of physical activity should be provided each day during schooltime, to help children achieve the physical activity recommendations^8^. In 2012, The Daily Mile was introduced as a physical activity intervention in primary schools, which was deemed easy to implement and accessible for all^9^. As part of the intervention, children and teachers leave the classroom each day to complete 15-min of movement outdoors, irrespective of the weather. To date (as of January 2022), ~ 13,790 nurseries and schools participate in the Daily Mile, with the widespread implementation success attributed to teacher autonomy and the flexibility this allows in terms of intervention delivery^10,11^. Despite the implementation success, there are concerns as to whether The Daily Mile improves physical fitness, body composition and cognition in children, as it is suggested a compensatory reduction in physical activity can occur across the school day after the implementation of a physical activity intervention such as The Daily Mile^12^. Therefore, research examining the chronic effect of The Daily Mile on physical activity levels, physical fitness, body composition and cognition in children is essential to ensure the intervention improves lifestyle risk factors (such as physical fitness and body composition) and cognitive function.

To date, the effect of school-based physical activity interventions on cognitive function in children (aged 7–12 years) has been difficult to determine due to the discrepant findings of previous research^13,14^. For example, a 20-week classroom-based and active transport intervention in children aged 7–9 years, did not improve executive function (measured via the Flanker task) or mathematic skills, despite improvements in physical fitness and body composition^14^. In contrast, a 36-week after-school, intermittent, moderate-to-vigorous physical activity intervention in children (aged 12 years), improved executive function (again measured via the Flanker task) and cognitive flexibility (measured via the colour-switch task)^13^. Consistent with the findings of Tarp et al.^14^, the intervention enhanced physical fitness and reduced body composition (body mass index; BMI) in the intervention group, which was associated with the cognitive improvements reported by Hillman et al.^13^. However, the discrepant findings of previous research might relate to differences in the intensity and duration of the physical activity interventions examined, the age of the young people recruited to the study and the potential that the physical activity intervention might have been offset with compensatory reductions in physical activity elsewhere across the day^12^. Irrespective of the reasons why previous research has found conflicting effects of school-based physical activity interventions on cognitive function, a recent review concluded that physical activity has a beneficial effect on cognitive outcomes in young people^15^. The potential benefits of physical activity for cognition further highlights the need for research that examines whether The Daily Mile, the most popular school-based physical activity intervention, has beneficial effects on cognitive function. This is particularly important given the associations between cognitive function and academic achievement^6,7^, and the emphasis that schools place upon academic achievement.

There is currently a paucity of research examining the effects of The Daily Mile on cognitive function in children, with only three studies to date examining the acute effects on different domains of cognition^16–18^. Early findings suggest that an acute bout of The Daily Mile does not improve working memory (measured via the Sternberg Paradigm and Digit Recall Test), inhibitory control (measured via the Stroop Test) or cognitive flexibility (measured via the Flanker Task and Trail Making Task) in children aged 8–11 years^15,16^. However, utilising a citizen science approach in a large cohort of primary school children in the UK (n = 5463), completing 15 min self-paced exercise (as is the case in The Daily Mile) enhanced inhibitory control, visual-spatial working memory and verbal working memory, when compared to both a resting control and more intense physical activity^18^. These discrepant findings may be explained by a lack of control over some key experimental procedures using a citizen science approach, as highlighted by the authors^18^.

The discrepant findings of previous research might be of concern to teachers and school leaders who may have implemented The Daily Mile for immediate improvements in cognition during the school day. Furthermore, a follow-up of the large citizen science study revealed no differences between those young people who regularly complete The Daily Mile, compared to those who do not^19^. However, an important secondary finding of the citizen science study^19^ and that of Hatch et al.^15^ was that children with higher physical fitness (measured as distance ran on the multi-stage fitness test; MSFT) displayed enhanced cognition compared to less fit children. Therefore, given the positive association between physical fitness and cognitive function in children^5,16^, and the potential for participation in The Daily Mile to enhance fitness^19^, it is important future research concurrently examines the chronic effects of The Daily Mile on physical fitness and subsequent cognitive function, as it might be that the benefits on cognition occur chronically via improvements in physical fitness.

Finally, improvements in physical fitness and body composition are not only important for enhancing cognitive function, but also for the associated enhancement of cardiometabolic health in children and adolescents^4,5^. Chronic participation (28-week intervention) in The Daily Mile has been reported to improve physical fitness (distance ran on the MSFT) and adiposity (sum of four skinfolds) in children aged 8 years^20^. However, the findings of this study have come under scrutiny given several limitations in the study design, relating to differences in exposure (intervention group completed 28-weeks of The Daily Mile, whilst the control group completed pre- and post-measurements with an interval of only 12-weeks), seasonal variations in the start and end times across intervention and control groups, and small sample sizes across just two primary schools in a quasi-experimental study design^21^. Nonetheless, as the authors highlight in their response^22^, the potential positive effects of The Daily Mile on body composition and physical fitness are of interest and relevance to those interested in implementing the initiative.

More recent studies have examined the effect of chronic participation of The Daily Mile on adiposity but have found discrepant findings. For example, whilst 12-months participation in The Daily Mile attenuated an increase in BMI z-scores in girls^23^, other studies have reported no effect on BMI from 12 or 24-weeks participation in The Daily Mile^24^. It is possible that such null findings might relate to the study design, whereby The Daily Mile was only completed on 2–3 days per week and not the intended 5 days^9^. Future research that addresses these limitations is necessary to determine the chronic effects of The Daily Mile on physical fitness and adiposity in primary school children.

Therefore, the aim of the present study was to examine the effect of 5 weeks participation in The Daily Mile on the cognitive function (attention, information processing, inhibitory control and working memory), physical fitness and adiposity in primary school children.

## Methods

### Participant characteristics

Seventy-nine children from years four, five and six (aged 10.3 ± 0.8 years) were recruited from two local primary schools (who had not previously implemented The Daily Mile) to participate in this quasi-experimental study, whereby one class from each school would complete either the exercise intervention (The Daily Mile; n = 44) or act as the control group, who continued their habitual activities (n = 35). A power calculation indicated that for the present study design and an effect size of 0.2 (reflective of the literature e.g. Ref.^25^), with two groups and two measurement points (α = 0.05), that a sample size of 68 was required. During familiarisation, participant body mass (Seca 770 digital scale, Hamburg, Germany), stature and sitting stature (Leicester Height Measure, Seca, Hamburg, Germany) were measured and subsequently used to calculate body mass index (BMI).

### Study design

Ethical approval was received from the Nottingham Trent University Ethical Advisory Committee (application 692), with all methods undertaken thereafter performed in accordance with the relevant guidelines and regulations. Headteacher approval for the study to commence and written informed parental/guardian consent were obtained. Parents/guardians also completed a health screen questionnaire on behalf of their child/dependent, which was checked by a lead investigator to ensure there were no medical conditions (such as a congenital heart condition, or a blood-line relative that had died during or soon after exercise) that would prevent the participant from completing the study. Participant assent (willingness to participate) was also obtained.

**Figure 1:**
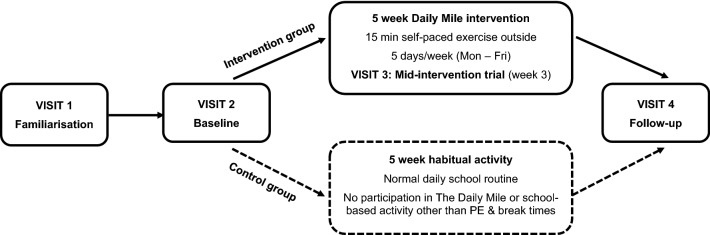
Overview of study design.

Participants completed three data collection visits; including a familiarisation session, a baseline trial, and a follow-up trial (see Fig. 1 for protocol schematic). In between baseline and follow-up trials, the intervention group completed 5 weeks of daily participation in The Daily Mile, whilst the control group continued with their normal daily school routine. Follow-up data collection was completed within 48 h of the final training session completed by the intervention group. Data collection was completed across two primary schools, the first completing the study between May–July 2021, and the second between October–December 2021. Each school had an intervention and a control group for whom baseline and follow-up testing occurred simultaneously, thus controlling for seasonal variations and overcoming limitations of previous work^18^.

### Experimental visits

#### Familiarisation

During the familiarisation session, participants were introduced to all the procedures involved in the present study and were provided with the opportunity to ask any questions they had in relation to the study protocol. Participants were familiarised with The Daily Mile (to ensure they understood the core components of the physical activity; Ref.^9^) and the battery of cognitive function tests, which were practised twice to minimise any potential learning effects.

#### Baseline and follow-up trials

During the 24 h prior to baseline and follow-up trials, participants were asked to refrain from strenuous physical activity and to record a food diary so that the same diet could be consumed prior to each assessment (baseline and follow-up). Participants were also asked to arrive to school following an overnight fast from 9 pm the previous evening, with only water allowed to be consumed ad libitum during this time. Parents/guardians were contacted the evening prior to baseline and follow-up trials to remind them of the pre-testing requirements. The follow-up trial was scheduled 48 h after the final Daily Mile session in the intervention group.

**Figure 2:**
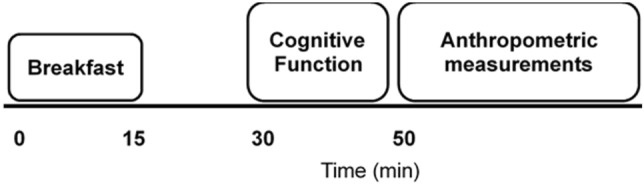
Timeline of measurements for baseline and follow-up trials.

Upon arrival to school, participants were provided with a standardised breakfast (cornflakes, milk, and toast with butter), which provided 1.5 g carbohydrate per kg body mass (as previously used, e.g.,^26,27^). Participants were allowed 15-min to consume breakfast, with water allowed ad libitum throughout. Thereafter, participants completed the battery of cognitive function tests, which commenced 30-min after the start of breakfast (for study timeline, see Fig. 2). After the cognitive function test battery was complete, waist circumference, hip circumference and four skinfolds were taken for the assessment of body composition. On a separate day, within 48 h, participants completed the multi-stage fitness test (MSFT) as a measure of cardiorespiratory fitness.

### Experimental procedures and measurements

#### Battery of cognitive function tests

The battery of cognitive function tests included the Stroop test (measure of information processing and inhibitory control), Sternberg paradigm (measure of visual working memory) and Flanker task (measure of attention and inhibitory control), which were completed in that order. Full details of each of these cognitive tests and are provided elsewhere^16^, which have previously been used successfully in a similar study population^16^ and the reliability reported^28^. The test battery was completed 30-min after the start of breakfast (Fig. 2) and lasted ~ 15-min. All tests were administered via a laptop computer (Lenovo ThinkPad T450, Lenovo, Hong Kong), with participants wearing noise cancelling headphones. During the cognitive function tests, participants also sat separately from one another and in a dimmed room, to minimise distractions. Verbal instructions of each test were provided by a lead investigator, which were followed by written instructions on screen for participants to read. Participants were reminded at the start of each test to answer correctly and as quickly as possible. Questions were encouraged and following confirmation all participants understood the test requirement, the battery of tests were undertaken.

Each test within the battery commenced with 3–6 practice stimuli, with feedback provided relating to whether the correct answer had been chosen. The practice stimuli allowed re-familiarisation with the test to further remove any potential learning effects. Outcome variables for each of the tests was the percentage of correct responses (%) and the response time (ms) to achieve each correct response. Each of the cognitive function tests included in the battery have previously been used successfully in a similar study population^16^.

#### Body composition measurements

For the assessment of body composition, participants had waist circumference, hip circumference, and four skinfold sites measured. Waist circumference was measured with a tape measure at the narrowest point between the xiphoid process and the iliac crest, whilst hip circumference was measured at the greatest diameter of the hip (both accurate to 0.1 cm). The four skinfold sites included the tricep, subscapular, supraspinale and front thigh and were taken in accordance with The International Society for the Advancement of Kinanthropometry guidance by trained kinanthropometrists. Waist circumference, hip circumference and each skinfold were measured twice and the mean value used as the criterion measure. In the event that the initial two measurements were discrepant by > 5%, a third measure was taken and the median used as the criterion value. In addition, from body mass and stature BMI (body mass/height^2^) and BMI percentiles (based on age and sex-specific British 1990 growth reference data^29^ were calculated.

#### Multi-stage fitness test

Distance ran on the multi-stage fitness test (MSFT;^30^) was the chosen measure of cardiorespiratory fitness, as previously used in a similar study population^16^. In brief, participants completed 20-m progressive shuttle runs to the point of volitional exhaustion. The test commenced at a speed of 8.0 km h^−1^ and then increased by 0.5 km h^−1^ for each subsequent 1-min stage completed. During the test, participants were paced by an experienced member of the research team and verbal encouragement was provided to ensure participants reached the point of volitional exhaustion.

### The Daily Mile

The Daily Mile requires participants to complete 15-min of informal, self-paced, outdoor physical activity around a pre-determined route^9^. During the exercise intervention, participants in the intervention group completed The Daily Mile every day (Monday through Friday) for 5 weeks. The classroom teacher was responsible for administering The Daily Mile, in line with recommendations from The Daily Mile (to be completed each day at their chosen time, irrespective of the weather). Teacher implementation logs were recorded daily during the intervention. For any days The Daily Mile was not completed, information relating to the reasons for this were recorded. Fidelity (number of days completed/number of days available for implementation × 100; %) was recorded for each participant, as well as for the experimental group overall. Whilst the intervention group completed five weeks of The Daily Mile, the control group continued with their normal school routine and habitual physical activity.

### Statistical analysis

All cognitive data were initially attended to in the open source software R (www.r-project.org). Minimum (< 100 ms) and maximum (1000–4000 ms depending on task complexity) response time cut-offs were applied to eliminate any unreasonably fast (anticipatory) or slow (distracted) responses, and the distribution of remaining cognitive data were assessed. This method has previously been used in similar studies^16,26,31,32^. Statistical analyses were then performed using Statistical Package for the Social Sciences (SPSS; Version 26; SPSS Inc., Chicago, IL., USA). Analysis of covariance (ANCOVA) was used for all outcome variables, to examine the between group (intervention vs. control) differences at follow-up, while controlling for the baseline score (covariate) of that outcome. This approach is considered most appropriate and is recommended for experimental designs such as the one used in the present study^33^. For all variables, the mean and standard deviation at baseline and follow-up for each group are presented, as well as the adjusted follow-up means and 95% CI; effect sizes are calculated as Cohen’s d and interpreted as per convention (0.2 = small, 0.5 = medium, 0.8 = large). Statistical significance was accepted as *p* < 0.05.

## Results

### Intervention fidelity

All but seven participants from the intervention group completed The Daily Mile every school day (5 days per week) for 5 weeks, totalling 25 days of participation. The seven participants that did not participate in all 25 days failed to do so due to being absent from school with illness. The number of days missed by these participants ranged from 1 to 11 days and thus fidelity in these participants ranged from 56 to 96%. Average fidelity of the intervention group as a whole, including the participants who were not able to attend every session, was 24 ± 4 days, which is equal to 96 ± 13%.

### Cognitive function

Response time and accuracy data, for each of the cognitive function tests, at baseline and follow-up, in both the intervention and control groups can be found in Table 1.

**Table 1:** Cognitive function data for the intervention and control groups at baseline and follow-up. Data are mean ± SEM.

Test	Variable	Test level	Intervention group		Control group		Adjusted follow-up mean [95% CI]			Cohen’s *d*
			Pre	Post	Pre	Post	Intervention	Control	*p* value	
Stroop test	Response time (ms)	Simple	1124 ± 42	1116 ± 57	1058 ± 43	1027 ± 55	1084 [1011, 1158]	1060 [985, 1136]	0.653	0.08
		Complex	1500 ± 58	1399 ± 58	1385 ± 60	1419 ± 59	1357 [1280, 1400]	1463 [1410, 1523]	0.048*	**0.31**
	Accuracy (%)	Simple	98.6 ± 0.8	97.5 ± 0.9	96.2 ± 0.8	97.1 ± 0.9	96.8 [95.2, 98.4]	97.8 [96.1, 99.4	0.434	0.20
		Complex	95.8 ± 1.4	95.0 ± 1.8	90.9 ± 1.4	90.6 ± 1.9	93.5 [90.2, 96.8]	92.2 [88.7, 95.6]	0.580	0.14
Sternberg paradigm	Response time (ms)	One-item	736 ± 30	676 ± 26	704 ± 31	686 ± 27	667 [629, 7.4]	696 [657, 735]	0.282	0.17
		Three-item	896 ± 31	828 ± 31	887 ± 31	867 ± 32	824 [781, 867]	870 [826, 914]	0.144	0.25
		Five-item	1054 ± 43	989 ± 44	1056 ± 44	1026 ± 45	990 [917, 1062]	1026 [951, 1100]	0.493	0.14
	Accuracy (%)	One-item	96.7 ± 1.0	93.8 ± 1.4	94.9 ± 1.0	96.5 ± 1.4	93.3 [90.7, 95.9]	97.0 [94.4, 99.6]	0.080	0.52
		Three-item	96.1 ± 0.7	95.4 ± 0.9	94.8 ± 0.8	95.4 ± 0.9	95.0 [93.4, 96.6]	95.8 [94.1, 97.5]	0.510	0.16
		Five-item	87.2 ± 2.1	87.3 ± 2.2	87.7 ± 2.2	88.5 ± 2.2	87.5 [83.9, 91.1]	88.4 [84.7, 92.1]	0.734	0.08
Flanker task	Response time (ms)	Congruent	782 ± 30	747 ± 31	722 ± 31	725 ± 32	726 [681, 772]	747 [700, 794]	0.541	0.11
		Incongruent	809 ± 34	789 ± 34	787 ± 35	788 ± 35	782 [730, 834]	795 [741, 849]	0.737	0.07
	Accuracy (%)	Congruent	95.6 ± 1.4	96.7 ± 1.0	95.1 ± 1.4	96.7 ± 1.0	96.6 [94.8, 98.5]	96.2 [94.3, 98.1]	0.768	0.06
		Incongruent	90.8 ± 2.5	91.9 ± 2.5	91.0 ± 2.6	91.9 ± 2.6	91.8 [88.7, 94.8]	91.9 [89.0, 94.9]	0.943	0.01

Significant values are in bold.

*Significant difference between intervention and control group at follow up (*p* < 0.05).

#### Stroop test

For response times on the simple level of the Stroop test, there was no difference between the intervention and control group at follow-up (*p* = 0.653). However, for response times on the complex level of the Stroop test, the intervention group (1357 ms, 95% CI [1280 ms, 1400 ms]) were significantly faster at follow-up when compared with the control group (1463 ms, 95% CI [1410 ms, 1523 ms]) (*F*_(1,67)_ = 3.5, *p* = 0.048, *d* = 0.31).

For accuracy on the Stroop test, there was no difference between the intervention and control group at follow-up for either the simple (*p* = 0.434) or complex (*p* = 0.580) levels.

#### Sternberg paradigm

For response times on the Sternberg paradigm, there was no difference between the intervention or control group at follow-up on the one-item (*p* = 0.282), three-item (*p* = 0.144) or five-item (*p* = 0.493) levels. Similarly, there was no difference in accuracy at follow-up on any level of the Sternberg paradigm (one-item, *p* = 0.080; three-item, *p* = 0.510; five-item, *p* = 0.734).

#### Flanker task

Response times for both congruent (*p* = 0.541) and incongruent (*p* = 0.737) stimuli on the Flanker task were not different between the intervention and control group at follow-up. Similarly, accuracy was not different between the groups at follow-up (congruent: *p* = 0.768; incongruent: *p* = 0.943).

### Body composition

There were no differences in body mass (*p* = 0.319), BMI (*p* = 0.269), BMI centile (*p* = 0.385), waist circumference (*p* = 0.304), hip circumference (*p* = 0.519), or sum of skinfolds (*p* = 0.610), between the intervention and control group at follow-up (Table 2).

**Table 2:** Body composition and physical fitness for the intervention and control group at baseline and follow-up. Data are mean ± SD.

Variable	Intervention group		Control group		Adjusted follow-up mean [95% CI]			Cohen’s *d*
	Pre	Post	Pre	Post	Intervention	Control	*p* value	
Body mass (kg)	38.7 ± 9.6	39.1 ± 9.9	38.1 ± 9.6	38.5 ± 9.8	38.7 [38.5, 39.0]	38.9 [38.7, 39.1]	0.319	0.02
BMI (kg m^−2^)	18.1 ± 3.1	18.0 ± 3.1	18.3 ± 2.6	18.4 ± 2.7	18.1 [18.0, 18.3]	18.3 [18.1, 18.4]	0.269	0.07
BMI centile	58.8 ± 30.0	57.4 ± 30.4	61.7 ± 28.5	61.5 ± 29.5	58.7 [56.7, 60.7]	60.0 [57.8, 62.1]	0.385	0.04
Waist circumference (cm)	62.8 ± 8.6	62.7 ± 8.7	62.1 ± 9.8	63.5 ± 7.4	62.5 [60.8, 64.1]	63.8 [61.9, 65.6]	0.304	0.15
Hip circumference (cm)	77.1 ± 8.5	77.0 ± 8.5	77.6 ± 8.6	78.1 ± 8.9	76.9 [74.2, 79.6]	78.2 [75.2, 81.2]	0.519	0.15
Sum of skinfolds (mm)	33.2 ± 19.0	32.8 ± 20.0	34.8 ± 15.1	35.1 ± 14.6	33.5 [31.5, 35.5]	34.2 [32.0, 36.4]	0.610	0.04
MSFT distance (m)	720 ± 360	880 ± 360	720 ± 400	740 ± 340	880 [820, 940]	740 [680, 800]	0.002 *	**0.39**

Significant values are in bold.

*Significant difference between intervention and control group at follow up (*p* < 0.05).

### Physical fitness

There was a significant difference between the intervention and control group at follow-up for distance covered on the MSFT (Table 2). Specifically, the intervention group (880 m, 95% CI [820 m, 940 m]) ran further than the control group (740 m, 95% CI [680 m, 800 m]), indicative of greater physical fitness (*F*_(1,72)_ = 10.3, *p* = 0.002, *d* = 0.39).

## Discussion

The present study examined the effect of five weeks implementation of The Daily Mile on cognitive function, physical fitness, and body composition in children. The present study is the first to report improvements in executive function (inhibitory control, as evidenced by improved response times on the complex level of the Stroop test) and physical fitness (increased distance ran on the MSFT) following implementation of The Daily Mile in a randomised control trial design, when compared to a control group who continued with their normal school routine and habitual physical activity. In contrast, participation in The Daily Mile for 5 weeks did not affect working memory or attention, with no differences reported in accuracy or response times on either level of the Sternberg paradigm (working memory) or Flanker test (attention) at follow-up between groups.

The main finding of the present study was that following 5 weeks participation in The Daily Mile, response times on the complex level of the Stroop test were 7% quicker at follow-up in the intervention group compared with the control group. The quicker response times observed at follow-up in the intervention group were not at the expense of reduced accuracy and thus suggest an improvement in inhibitory control (as assessed by the complex level of the Stroop test), rather than a speed-accuracy trade off. Inhibitory control is a subset of executive function, which involves inhibiting dominant or automatic responses when necessary^34^. In children, executive dysfunction has been associated with poor classroom and academic outcomes, with impairments reported in reading comprehension, maths, and social relationships^34^. Therefore, the improvements observed in executive function after 5 weeks of The Daily Mile in primary school children have important implications for teachers and their teaching practice, providing them with the potential to improve academic outcomes through short-term (5 weeks) participation in The Daily Mile.

The improvements in executive function following the implementation of The Daily Mile in primary school children is a novel finding of the present study. However, this key finding is consistent with the limited previous research that has examined the effect of other school-based physical activity interventions on executive function in children^6,13^, yet is not in line with a large citizen science study that reported no differences in cognition between those young people who regularly complete The Daily Mile and those who do not^19^. Importantly, the present study is novel in that it specifically examined the effect of The Daily Mile using a randomised control design, rather than cross-sectionally comparing those who regularly do and do not complete The Daily Mile, as in previous work^19^; where confounding variables could influence study outcomes.

Understanding the impact of The Daily Mile on cognition is important because it is a much less demanding physical activity intervention in terms of the total daily exercise duration undertaken when compared with the duration of physical activity examined in previous research^6,13^; and is an intervention that has been implemented Worldwide. The Daily Mile consists of 15-min of daily physical activity in comparison with > 30-min of physical activity undertaken in earlier school-based research^6,13^. The beneficial effect of a shorter bout of daily physical activity on executive function in children, as observed in the present study, is of importance given that time is often cited as a barrier to school based physical activity interventions being implemented successfully long-term^35,36^. Furthermore, the present study examined the effect of short-term implementation of The Daily Mile, examining just 5 weeks of the intervention on cognitive function in children. Previous research that has reported improvements in cognition following a school-based physical activity intervention has examined longer duration interventions of > 20 weeks^6,13^. Thus, the present study provides details of the short timeframe in which improvements in cognitive function could be achieved in children following the implementation of The Daily Mile.

In contrast, 5 weeks of The Daily Mile had no effect on accuracy or response times on the Sternberg paradigm (a measure of working memory) or the Flanker test (a measure of attention) in primary school children. Tarp et al.^14^ also reported that a school-based physical activity intervention (specifically 20 weeks of classroom-based activities and active transport) had no effect on the attention of children aged 11–13 years, when attention was measured using the Flanker test. Whilst the findings of the present study, when taken in conjunction with those of Tarp et al.^14^ and the citizen science study of Booth et al.^19^, suggest that school-based physical activity interventions have no effect on attention or working memory, research in the field to date is limited and there are many variables relating to school-based physical activity interventions that need to be explored in the future. Therefore, future research should examine the effect of different physical activity modes, durations, intensities, and frequencies to optimise school-based interventions for the enhancement of all domains of cognitive function in primary school children, with the aim to enhance as many cognitive domains as possible. Furthermore, the sensitivity of the tests used to examine cognitive function in children might need to be reconsidered, as the Flanker task has repeatedly reported no effect of acute^16^ or chronic^14^ exercise on attention.

Physical fitness (as measured by distance ran on the MSFT) was significantly improved following The Daily Mile at follow-up when compared with the control group. Specifically, participants in the intervention group ran 19% further at follow-up when compared to the control group. Improvements in physical fitness have previously been reported by Chesham et al.^20^, whereby distance ran on the MSFT was enhanced in children following the implementation of seven months of The Daily Mile. The present study provides further novel evidence that the short-term implementation of The Daily Mile, over just 5 weeks, also improves the physical fitness of primary school children. This is of particular interest given the associations of physical fitness with cardiometabolic health^4,5^ and academic achievement during childhood^6,7^. However, as the present study did not directly examine the effect of The Daily Mile on cardiometabolic health and mental well-being in children, future research is warranted to ascertain whether The Daily Mile has further beneficial effects on childhood cardiometabolic and mental health. With regards the effect on physical fitness, this key finding has important implications as it highlights that physical fitness can be increased after just 5 weeks of The Daily Mile, which is a particularly relevant timeframe given each school half-term is between 5 and 7 weeks in duration in England.

Despite improvements in physical fitness, 5 weeks of The Daily Mile had no effect on the body composition (as measured by waist and hip circumference, BMI percentile, and the sum of four skinfolds) of primary school children in the intervention group when compared with the control group. The lack of an effect of The Daily Mile on body composition (measured as BMI) has previously been reported following 12 weeks and 24 weeks^24^ implementation in children aged 8–10 years. The lack of an effect of The Daily Mile on body composition in the present study might relate to the participant characteristics, with participants recruited to the intervention group, on average, being on the 59th percentile for BMI when compared with normative values for their age and sex^29^. As such, it could be argued that given the children recruited to the present study are overall classified as normal weight, there was limited potential for five weeks of The Daily Mile to reduce adiposity. Of importance is the potential for physical activity interventions to improve the body composition of children and adolescents who are classified as overweight (BMI ≥ 85th percentile) or obese (BMI ≥ 95th percentile), who are vulnerable to exposure of risk factors for many chronic non-communicable diseases, such as cardiovascular disease and type 2 diabetes^37^. Given the rising prevalence of overweight and obesity during childhood^38^, future research should examine the potential for The Daily Mile to improve the body composition of children categorised as overweight/obese.

The present study has several strengths, notably it is the first to examine the chronic effects of implementation of The Daily Mile on cognitive function in primary school children using a randomised crossover design. Furthermore, given the importance of acute nutrition (i.e., breakfast) on cognition in this population^39^ and the potential for breakfast and acute exercise to interact^40^ a key strength of the present study was the provision of a matched breakfast on each experimental trial. In addition, the residual effects of the final bout of The Daily Mile on cognition were minimised by allowing 48 h before the final measures were taken. However, the study is not without limitation. For example, whilst implementing The Daily Mile for a longer duration than previous studies (5 weeks), future work should examine the longer-term effects of implementation on cognition, physical fitness, and body composition, such as over an academic year. Furthermore, given the potential for cognition, physical fitness, and body composition to all influence each other^5^, the causal nature of these relationships warrants further investigation.

In conclusion, 5 weeks implementation of The Daily Mile improved inhibitory control and physical fitness in primary school children, yet had no effect on working memory, attention, or body composition. Therefore, the present study supports the wide-spread role out of The Daily Mile across schools and nurseries worldwide given the beneficial effects of chronic implementation on executive function and physical fitness in children. Furthermore, based on the improvements observed in executive function, teachers who are seeking methods to improve cognition through their teaching practice should consider implementing The Daily Mile during the school day. Furthermore, given the lack of an effect of The Daily Mile on body composition in the present study, future consideration should be given to the effect of The Daily Mile on adiposity in children who are categorised as overweight and obese, particularly as these children are at increased risk of exposure to risk factors for several non-communicable diseases, such as cardiovascular disease and type 2 diabetes. Finally, future research should continue to explore the effects of The Daily Mile on different domains of cognitive function, academic achievement and on the cardiometabolic and mental health of children.

## Data Availability

Original data are available from the corresponding author upon reasonable request.

## References

[1] UK Chief Medical Officers' Physical Activity Guidelines. Accessed Mar 2022. www.gov.uk/government/publications/physical-activity-guidelines-uk-chief-medical-officers-report (2022).

[2] Health Survey for England, 2019. Accessed Mar 2022. https://digital.nhs.uk/data-and-information/publications/statistical/health-survey-for-england/2019 (2022).

[3] Ortega FB (2010). Cardiovascular fitness modifies the associations between physical activity and abdominal adiposity in children and adolescents: The European Youth Heart Study. Br. J. Sports Med..

[4] Dring KJ (2019). Multi-stage fitness test performance, VO_2_ peak and adiposity: Effect on risk factors for cardio-metabolic disease in adolescents. Front. Physiol..

[5] Williams RA (2022). Physical fitness, physical activity and adiposity: Associations with risk factors for cardiometabolic disease and cognitive function across adolescence. BMC Pediatr..

[6] van der Niet AG, Hartman E, Smith J, Visscher C (2014). Modelling relationships between physical fitness, executive functioning, and academic achievement in primary school children. Psychol. Sport Exerc..

[7] Donnelly JE (2016). Physical activity, fitness, cognitive function, and academic achievement in children: A systematic review. Med. Sci. Sports Exerc..

[8] Public Health England, 2020. Accessed Mar 2022. https://assets.publishing.service.gov.uk/government/uploads/system/uploads/attachment_data/file/876242/Guidance_to_increase_physical_activity_among_children_and_young_people_in_schools_and_colleges.pdf (2020).

[9] The Daily Mile. The Daily Mile UK. Accessed Mar 2022. https://thedailymile.co.uk/ (2022).

[10] Ryde GC (2018). The Daily Mile: What factors are associated with its implementation success?. PLoS One.

[11] Marchant E, Todd C, Stratton G, Brophy S (2020). The Daily Mile: Whole-school recommendations for implementation and sustainability. A mixed-methods study. PLoS One..

[12] Love R, Adams J, van Sluijs EMF (2018). Are school-based physical activity interventions effective and equitable? A systematic review and meta-analysis of cluster randomised controlled trials. Lancet.

[13] Hillman CH (2014). Effects of the FITKids randomized controlled trial on executive control and brain function. Pediatrics.

[14] Tarp J (2016). Effectiveness of a school-based physical activity intervention on cognitive performance in Danish adolescents: Locomotion, learning, cognition and motion: a cluster randomized controlled trial. PLoS One.

[15] Chaput JP (2020). 2020 WHO guidelines on physical activity and sedentary behaviour for children and adolescents aged 5–17 years: Summary of the evidence. Int. J. Behav. Nutr. Phys. Act..

[16] Hatch LM (2021). The Daily Mile™: Acute effects on children’s cognitive function and factors affecting their enjoyment. Psychol. Sport Exerc..

[17] Morris JL, Daly-Smith A, Archbold VS, Wilkins EL, McKenna J (2019). The Daily Mile™ initiative: Exploring physical activity and the acute effects on executive function and academic performance in primary school children. Psychol. Sport Exerc..

[18] Booth JN, Chesham RA, Brooks NE, Gorely T, Moran CN (2020). A citizen science study of short physical activity breaks at school: Improvements in cognition and wellbeing with self-paced activity. BMC Med..

[19] Booth JN, Chesham RA, Brooks NE, Gorely T, Moran CN (2022). The impact of the Daily Mile on school pupils’ fitness, cognition and wellbeing: Findings from longer-term participation. Front. Psychol..

[20] Chesham RA (2018). The Daily Mile makes primary school children more active, less sedentary and improves their fitness and body composition: A quasi-experimental pilot study. BMC Med..

[21] Daly-Smith A, Morris JL, Hobbs M, McKenna J (2019). Commentary on a recent article on the effects of the ‘Daily Mile’ on physical activity, fitness and body composition: Addressing key limitations. BMC Med..

[22] Chesham RA (2019). Response to Daly_Smith et al.’s commentary on ‘The Daily Mile makes primary school children more active, less sedentary and improves their fitness and body composition: a quasi-experimental pilot study’. BMC Med..

[23] Breheny K (2020). Effectiveness and cost-effectiveness of The Daily Mile on childhood weight outcomes and wellbeing: A cluster randomised controlled trial. Int. J. Obesity..

[24] Brustio PR (2020). The Daily Mile is able to improve cardiorespiratory fitness when practiced three times a week. Int. J. Environ. Res. Pub. Health..

[25] Jackson WM, Davis N, Sands SA, Whittington RA, Sun LS (2016). Physical activity and cognitive development: A meta-analysis. J. Neurosurg. Anesthesiol..

[26] Cooper SB (2018). High intensity intermittent games-based activity and adolescents’ cognition: Moderating effect of physical fitness. BMC Pub. Health..

[27] Dring KJ (2020). Effect of exercise duration on postprandial glycaemic and insulinaemic responses in adolescents. Nutrition.

[28] Cooper SB, Bandelow S, Morris JG, Nevill ME (2015). Reliability of a battery of cognitive function tests in an adolescent population. J. Sport Sci..

[29] Cole TJ, Freeman JV, Preece MA (1995). Body mass index reference curves for the UK, 1990. Arch. Dis. Childhood..

[30] Ramsbottom R, Brewer J, Williams C (1988). A progressive shuttle run test to estimate maximal oxygen uptake. Br. J. Sports Med..

[31] Hatch LM (2021). Effect of differing durations of high-intensity intermittent activity on cognitive function in adolescents. Int. J. Environ. Res. Pub. Health..

[32] Williams RA (2020). Effect of football activity and physical fitness on information processing, inhibitory control and working memory in adolescents. BMC Public Health.

[33] Hecksteden A, Faude O, Meyer T, Donath L (2018). How to construct, conduct and analyze an exercise training study?. Front. Physiol..

[34] Gerst EH, Cirino PT, Fletcher JM, Yoshida H (2017). Cognitive and behavioral rating measures of executive function as predictors of academic outcomes in children. Child Neuropsychol..

[35] Malden S, Doi L (2019). The Daily Mile: Teachers’ perspectives of the barriers and facilitators to the delivery of a school-based physical activity intervention. BMJ Open.

[36] Naylor PJ (2015). Implementation of school based physical activity interventions: A systematic review. Prevent. Med..

[37] Pulgaron ER, Delamater AM (2014). Obesity and type 2 diabetes in children: Epidemiology and treatment. Curr. Diabetes Rep..

[38] Ogden CL (2016). Trends in obesity prevalence among children and adolescents in the United States, 1988–1994 through 2013–2014. JAMA.

[39] Cooper SB, Bandelow S, Nevill ME (2011). Breakfast consumption and cognitive function in adolescent school children. Physiol. Behav..

[40] Cooper SB, Bandelow S, Nute ML, Morris JG, Nevill ME (2015). Breakfast glycaemic index and exercise: Combined effects on adolescents’ cognition. Physiol. Behav..

