# Author Correction: Effect of 5-weeks participation in The Daily Mile on cognitive function, physical fitness, and body composition in children

**DOI:** 10.1038/s41598-022-22494-5

**Published:** 2022-10-21

**Authors:** Karah J. Dring, Lorna M. Hatch, Ryan A. Williams, John G. Morris, Caroline Sunderland, Mary E. Nevill, Simon B. Cooper

**Affiliations:** Sport Health and Performance Enhancement (SHAPE) Research Group, Department of Sport Science, School of Science and Technology, Nottingham Trent University, Clifton Campus, Nottingham, NG11 8NS UK

Correction to:* Scientific Reports*, 10.1038/s41598-022-18371-w, published on 22 August 2022

The original version of this Article contained an error in the spelling of one of the author names. Lorna M. Hatch was incorrectly given as Lorna A. Hatch.

The original Article has been corrected.

